# Differential Allocation to Photosynthetic and Non-Photosynthetic Nitrogen Fractions among Native and Invasive Species

**DOI:** 10.1371/journal.pone.0064502

**Published:** 2013-05-20

**Authors:** Jennifer L. Funk, Lori A. Glenwinkel, Lawren Sack

**Affiliations:** 1 School of Earth and Environmental Sciences, Chapman University, Orange, California, United States of America; 2 Department of Biological Sciences, Columbia University, New York, New York, United States of America; 3 Department of Ecology and Evolutionary Biology, University of California Los Angeles, Los Angeles, California, United States of America; University of Konstanz, Germany

## Abstract

Invasive species are expected to cluster on the “high-return” end of the leaf economic spectrum, displaying leaf traits consistent with higher carbon assimilation relative to native species. Intra-leaf nitrogen (N) allocation should support these physiological differences; however, N biochemistry has not been examined in more than a few invasive species. We measured 34 leaf traits including seven leaf N pools for five native and five invasive species from Hawaii under low irradiance to mimic the forest understory environment. We found several trait differences between native and invasive species. In particular, invasive species showed preferential N allocation to metabolism (amino acids) rather than photosynthetic light reactions (membrane-bound protein) by comparison with native species. The soluble protein concentration did not vary between groups. Under these low irradiance conditions, native species had higher light-saturated photosynthetic rates, possibly as a consequence of a greater investment in membrane-bound protein. Invasive species may succeed by employing a wide range of N allocation mechanisms, including higher amino acid production for fast growth under high irradiance or storage of N in leaves as soluble protein or amino acids.

## Introduction

The invasion of non-native species poses a rapidly growing threat to biodiversity and ecosystem function in many parts of the world, a threat equal to or stronger than that of climate change [1]. Thus, the need to understand the mechanisms of invasion has never been greater. Recent work has suggested that native and invasive plant species have similar carbon capture strategies but are aligned on opposite sides of a leaf economics spectrum [2]. For example, while native and invasive species show similar relationships among variables such as leaf nitrogen (N) concentration, photosynthetic rate (A), and leaf mass per unit area (LMA), invasive species cluster on the “high-return” end of the leaf economics spectrum with higher values of leaf N and A and lower values of LMA relative to natives [3], [4]. Many of these traits, such as LMA, leaf N and A, correlate with relative growth rate [5], [6], but see [7], [8] and higher relative growth rates facilitate invasion in some systems [9], but see [10]. Intra-plant N allocation should reflect these trait differences [11]; however, the biochemical basis underlying these patterns has not been examined in native and invasive species.

Within leaves, N occurs in soluble and membrane-bound proteins which are involved in carbon assimilation and light harvesting, respectively, as well as non-photosynthetic compounds such as cell wall proteins, amino acids, nucleic acids, defense compounds (e.g., alkaloids), and inorganic N (NO_3_^−^, NH_4_^+^). Plant species vary greatly in how N is allocated among these pools. For example, plants may allocate 5–32% of leaf N to Rubisco and 2–30% to cell walls, with higher amounts of cell wall protein in longer-lived leaves [12]–[17]. Notably, studies examining intra-leaf N pools have tended to focus primarily on cell-wall protein as the primary non-photosynthetic fraction without quantifying N allocation to amino acids, nucleic acids, inorganic N and secondary N-containing compounds [14]–[16], [18], [19], but see [20], [21]. This level of resolution may be critical for determining important traits of invasive species. If invasive species occupy the “high-return” of the leaf economics spectrum with high A and low LMA, they should allocate more N to photosynthetic enzymes, and amino acids and nucleic acids as precursors of protein synthesis and growth. It is also possible that allocation of N to amino acids and nucleic acids (required for protein synthesis and growth) may have an equal or stronger effect on plant performance than increasing the amount of soluble protein involved in photosynthesis [21]–[22]. This hypothesis is supported by many studies where fertilization of woody species (primarily fast-growing forestry species) resulted in small changes in leaf N, but large changes in relative growth rate [23]–[29]. Conversely, if native species occupy the “slow-return” end of the leaf economics spectrum, requiring tougher, longer-lived leaves for a positive carbon balance, these species should allocate more N to structure (cell-wall protein) at the expense of metabolic processes such as photosynthesis and growth.

The goal of this study was to examine intra-leaf N pools within a suite of native and invasive species and determine how these N pools correlate with leaf-level morphological and physiological traits, such as A and LMA. We characterized 34 leaf traits, and in particular leaf N partitioning with a high level of resolution, in five native and five invasive woody species that co-occur in Hawaii. All species establish on young, N-poor volcanic soils ranging in age from 100 to 10,000 years with ample water availability [30]; thus, plant growth is limited primarily by N availability in open areas, and co-limited by N and irradiance under closed canopies. While relative growth rates were not directly measured for these species, data collected in our study demonstrated that the invasive species selected generally had lower LMA than natives, aligning them closer to the “high-return” end of the leaf economics spectrum.

We examined seven intra-leaf N pools including soluble protein, membrane-bound protein, cell-wall protein, total nucleic acid, amino acid and inorganic N (NO_3_^−^, NH_4_^+^). The soluble protein fraction contains Calvin cycle and photorespiratory enzymes of which up to 50% is Rubisco [12]. All ten species were grown under shaded conditions (300 µmol photon m^−2^ s^−1^ at mid-day) to simulate natural conditions for seedlings establishing in the understory of native mesic forests in Hawaii (dominated by *Metrosideros polymorpha*). Previous theoretical estimates based on gas exchange data have indicated that shade leaves should have a 1∶1 ratio of soluble to membrane-bound protein [12], [31]. We expected that invasive species would have higher A and lower LMA and, consequently, would allocate more N to soluble protein, amino acids and nucleic acids and less N to cell wall protein.

## Materials and Methods

### Plant material

Seeds were collected on the Island of Hawaii in November 2007 for the native species *Acacia koa* (Fabaceae), *Dodonaea viscosa* (Sapindaceae), *Osteomeles anthyllidifolia* (Rosaceae), *Pipturus albidus* (Urticaceae) and *Sophora chrysophylla* (Fabaceae), and for the invasive species *Falcataria moluccana* (Fabaceae), *Leucaena leucocephala* (Fabaceae), *Psidium cattleianum* (Myrtaceae), *Pyracantha angustifolia* (Rosaceae), and *Schinus terebinthifolius* (Anacardiaceae); nomenclature follows Wagner et al. [32]. All necessary permits were obtained for the described field studies (Tim Tunison, Hawaii Volcanoes National Park). All ten species are woody shrubs or trees with C_3_ photosynthesis. All five invasive species are considered high risk weeds (A Global Compendium of Weeds, http://www.hear.org/gcw/index.html). Only one of the five native species (*Dodonaea*) is indigenous or naturalized outside of Hawaii. Because many of the worst invaders in Hawaii are legumes, we included two invasive (*Falcataria, Leucaena*) and two common native (*Acacia, Sophora*) legume species.

All non-legumes co-occur within Hawaii Volcanoes National Park and were chosen based on abundance, growth habit, and viability of seed. Seeds were germinated in potting soil in March 2008 and grown at the UCLA Plant Growth Facility greenhouse. After 3–6 months, plants were transferred to 3.6 L pots to avoid pot-binding. All plants received fertilization (0.2 mg) during irrigation every two days [Scotts Peters Professional water soluble fertilizer, N-P-K ratio 20-20-20; N as NH_4_^+^ (4.1%), NO_3_^−^ (5.5%), urea (10.4%); P as P_2_O_5_; K as K_2_O]. Pots were watered to field capacity. Irradiance levels within the greenhouse ranged from 60–550 µmol photon m^−2^ s^−1^, with an average value of 300 µmol photon m^−2^ s^−1^ at mid-day. Our efforts focused on intensive physiological and biochemical measures and relative growth rate data were not collected in this study.

### Gas exchange measurements

In January 2009, photosynthetic rates and chlorophyll fluorescence were measured with a LI-6400 portable photosynthesis system with a fluorescence chamber (LI-COR, Lincoln, NE, USA). All physiological and biochemical measures were conducted on fully-expanded, recently mature leaves. We conducted light response curves on two individuals per species and determined that 1000 µmol photon m^−2^ s^−1^ was a saturating irradiance for all species. We conducted all measures at this irradiance. Relative humidity was maintained between 40–60% and ambient temperature was 25°C.

The effective quantum yield of PSII (φ_PSII_), measured at 1000 µmol photon m^−2^ s^−1^, was calculated as (F_m_′-F_s_)/F_m_′, where F_s_ is the fluorescence yield of a light-adapted leaf and F_m_′ is the maximal fluorescence during a saturating light flash. CO_2_-response curves were determined by varying chamber CO_2_ concentration between 50 and 2000 µL L^−1^ (50, 100, 200, 400, 600, 1000, 1500, 2000). Maximum carboxylation rate (V_max_) and maximum photosynthetic electron transport rate (J_max_) were estimated from the CO_2_-response curves based on [33], [34] using temperature responses outlined in ref. [35].

Following gas exchange measures, we measured leaf thickness with digital calipers at three points along the leaf to account for variation in thickness. Leaves were then harvested, scanned for leaf area, dried, and weighed to determine leaf mass per unit area (LMA). Leaf density (g cm^−3^) was calculated by dividing LMA by leaf thickness. Total leaf N concentration was determined with an elemental analyzer (CE Instruments Flash EA 1112, CE Elantech). All physiology measurements were conducted on five individuals per species.

### Biochemical measurements

Leaves adjacent to gas exchange leaves were harvested, immersed in liquid nitrogen and stored at −80°C prior to processing. We pulverized 30 to 100 mg of leaf tissue in liquid nitrogen prior to each extraction procedure. All reagents and solutions were made with ultrapure water (Barnstead NANOpure InfinityTM Ultrapure Water System. Prod. >17.00 MΩ −CM). Pigments were isolated in acetone and determined colorimetrically with a UV/VIS spectrophotometer (Beckman DU-640) following the methods of [36]. Chlorophyll a and b concentration were determined using a multi-wavelength analysis at 645, 662, and 710 nm. We did not have enough tissue to perform pigment analysis on *Acacia koa*.

Water-soluble, SDS-soluble, and SDS-insoluble protein fractions were extracted using a modified version of the procedure described in ref. [16] in which the SDS-soluble fraction consists of membrane-bound proteins and the SDS-insoluble fraction consists of cell-wall proteins. Previous work has shown a tight correlation between soluble protein and Rubisco concentration with Rubisco accounting for approximately 50% of the soluble protein fraction [31]. We used 4% polyvinylpolypyrrolidone (PVPP, or cross-linked insoluble PVP) in place of PVP with a 100 mM Tris Extraction buffer, pH 7.4 for the effective absorption of polyphenols [37]. The pH of 7.4 allows for maximum extraction efficiency combined with maximum polyphenol absorption by PVPP [38] and significantly increases water soluble protein yields [39]. While this protein fractionation method has been used in a diverse array of studies, e.g., [16], [18], the method underestimates soluble and membrane-bound protein fractions when phenolic compounds are present. We corrected for the interference by phenolics for species with total phenolic concentration greater than 12% dry leaf mass (*Pipturus, Psidium*, and *Schinus*) (see Methods S1). Our correction resulted in a +/−20% error for protein fractions in these three species. This error is similar to or smaller than that associated with calculations of intra-leaf N partitioning which rely on estimates of Rubisco activity and bioenergetics derived from gas exchange measures [14], [40]–[45].

The three protein fractions were hydrolyzed with barium hydroxide [46] and then quantified using an updated version of the ninhydrin method [47]. Because the ninhydrin method is poor for detecting hydroxyproline or proline [16], which are abundant in cell-wall proteins [17], this assay likely underestimates the amount of structural protein. However, this method is appropriate for assessing the relative differences in protein fractions among species. We used bovine serum albumin for standards.

Nucleic acids were extracted using a modified version of the method in ref. [20]. Samples were first extracted in 2 ml of chloroform: methanol (2∶1). Samples were then sequentially extracted twice with chloroform: methanol: water (1∶1∶0.8), once with cold 80% methanol, and once with cold 5% trichloroacetic acid (TCA). All sequential extractions were performed for 15 min with 1 ml of extraction medium. After each extraction the samples were centrifuged at maximum speed for 2 minutes and the supernatant discarded. The final pellet was extracted twice with 2.5% TCA for one hour at 95°C. For total nucleic acid-nitrogen (TNA) quantification, the supernatant containing TNA was digested using the persulfate digest method [48] with 4.9 ml of water, 100 µl of TNA extract, and 666 µl of persulfate reagent. Each reaction vial was autoclaved at 120°C for 1.5 h [49], cooled at 4°C and buffered with 1 ml of 0.7 M ammonium chloride, pH 8.5 [48]. We added 0.5 to 0.6 g of spongy cadmium to each reaction tube and placed vials on a shaker table in the dark for 1.5 hours. We then added 250 µl of color reagent [48] and allowed the samples to develop for 10 minutes in the dark. We read the absorbance at 540 nm using a microplate reader. Potassium nitrate and calf thymus DNA (Sigma Aldrich D4522) were used as standards.

Amino acids were extracted with 80% methanol [50] and quantified using the Ninhydrin method [47], modified for use with a microplate reader. We added 120 µl of ninhydrin solution to 120 µl of amino acid extract. The samples were then incubated at 100°C for 15 minutes and cooled on ice. We added 440 µl of cold 50% isopropanol to each sample and read the absorbance at 570 nm. Ammonium was extracted as in ref. [51] using 2 ml of 80% methanol and quantified by fluorimetry using HPLC. Nitrate was extracted as in ref. [52] and absorbance was read at 405 nm using a microplate reader.

### Statistical analysis

To determine differences in biochemical and gas exchange traits among five native and five invasive species, we used a nested ANOVA with ‘origin’ (native and invasive species) as a fixed effect and ‘species’ nested within origin. To determine differences in biochemical and gas exchange traits among legumes and non-legumes, we used a nested ANOVA with ‘legume’ as a fixed effect and ‘species’ nested within legume. Data that violated the ANOVA assumptions of normality and homogeneity of variance were Box–Cox transformed.

For traits that correlated with leaf mass per area (LMA), we tested whether the differences between native and invasive species could be explained by differences in LMA. We tested the trait-LMA relationships between native and invasive species for differences in slope and intercepts using SMATR (http://www.bio.mq.edu.au/ecology/SMATR/index.html; [53]). For this test, we used raw and log-transformed data (i.e., fitting a power-law relationship).

A multivariate analysis was conducted on biochemical traits using principal components analysis (PCA). All traits were standardized prior to analysis using the formula [(trait – trait mean)/trait SD]. Pearson product–moment correlation coefficients were generated to evaluate the linear association among biochemical, physiological and morphological traits. All ANOVA, PCA and correlation analyses were performed in JMP 8 (SAS Institute, Inc., Cary, NC, U.S.A.).

## Results

### Nitrogen fraction recovery

Across species, we recovered between 38 to 108% of total leaf N in our seven fractions (soluble protein, membrane-bound protein, cell-wall protein, total nucleic acid, amino acid, NH_4_^+^, and NO_3_^−^, see Results S1). Lower recovery in some species could be due to the presence of nitrogenous compounds not measured (e.g., alkaloids) or interference during N fraction extraction. These deviations did not occur in any systematic way; that is, the sum of N fractions was not substantially lower at low or high leaf N, for native versus invasive species, or for legumes versus non-legumes (see Results S1). For consistency across the results, we present the percentage of leaf N allocated to various N fractions using the value obtained by summing our seven fractions rather than that obtained by elemental analysis. Throughout, we use the term ‘concentration’ to refer to a parameter expressed per unit dry mass and ‘content per area’ for the area basis.

### General patterns across species

**Figure 1:**
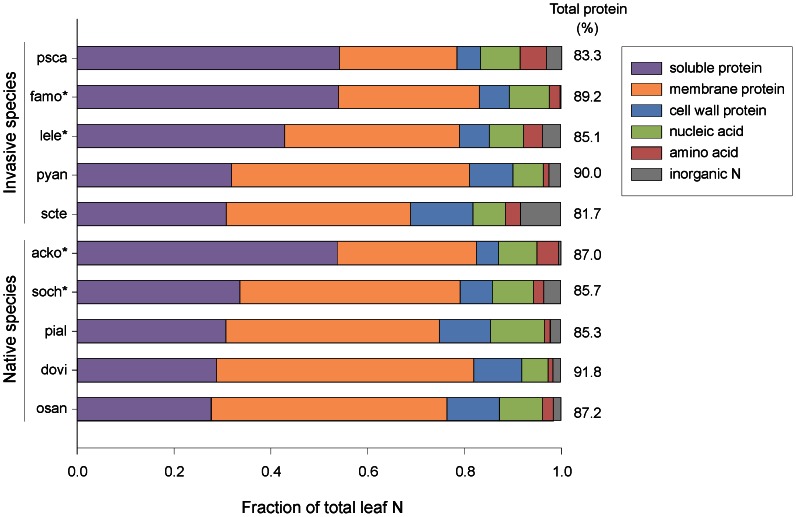
The fraction of total leaf N present in six biochemical fractions for five native and five invasive species grown at 300 µmol photon m^−2^ s^−1^. The percent of total leaf N represented by all three protein fractions is provided on the right. Inorganic N includes nitrate and ammonium. Species abbreviations are: *Acacia koa* (acko), *Dodonaea viscosa* (dovi), *Falcataria moluccana* (famo), *Leucaena leucocephala* (lele), *Osteomeles anthyllidifolia* (osan), *Pipturus albidus* (pial), *Psidium cattleianum* (psca), *Pyracantha angustifolia* (pyan), *Schinus terebinthifolius* (scte), and *Sophora chrysophylla* (soch). Legumes are marked with an asterisk (*).

The allocation of leaf N to protein (81.7 to 91.8%) varied relatively narrowly across species (Figure 1). However, species varied substantially in their distribution of protein among soluble, membrane-bound and cell-wall fractions. Total nucleic acid and amino acid concentrations and contents per area varied significantly among species (Figure 1). The fraction of leaf N allocated to nucleic acid ranged twofold from 5.4 to 11.2% across species while the allocation to total amino acid concentration ranged fivefold from 1.1 to 5.4% (Figure 1). Variation among species in inorganic N was driven by a few species as discussed below.

### Variation in nitrogen partitioning between native and invasive species

Invasive species differed from natives in several leaf physiological and biochemical traits (Table 1). Invasive species had lower LMA and, consequently, lower leaf N content per area compared to native species. Invasive species also had lower photosynthetic function per area (A_area_, V_max_, J_max_, Table 1) compared to native species. With respect to biochemical traits, native species had high absolute values of membrane-bound, cell-wall, and total protein concentrations and content per area compared to invasive species as well as a higher fraction of total leaf N allocated to membrane-bound protein (Table 1). Soluble protein concentration, content per area, and the ratio of soluble to membrane-bound protein (invasive, 1.33; native, 1.00) did not differ significantly between native and invasive species (Table 1). Invaders allocated a higher percentage of leaf N to total amino acid and NH_4_^+^ compared to native species, driven by higher NH_4_^+^ in *Psidium* and *Schinus* (Table 1, Table S1). Chlorophyll concentration and chlorophyll content per area were similar between native and invasive species.

**Table 1:** Group means (and standard error in parentheses) for invasive and native species, and for legumes and non-legumes grown at 300 µmol photon m^−2^ s^−1^.

	Native species (n = 5)	Invasive species (n = 5)	Effect of origin	Legumes (n = 4)	Non-legumes (n = 6)	Effect of legume
**Physiology**						
A_area_	12.5 (0.7)	10.5 (0.6)	5.13*	11.8 (0.6)	11.3 (0.6)	0.49
A_mass_	277 (31)	295 (28)	0.17	335 (30)	254 (29)	3.81
V_max_	100.9 (5.4)	78.7 (5.0)	11.09**	106.8 (5.1)	78.4 (5.1)	17.65**
J_max_	173 (9)	131 (9)	11.60**	171 (9)	139 (9)	8.70**
φ_PSII_	0.23 (0.01)	0.21 (0.01)	1.55	0.24 (0.01)	0.20 (0.01)	4.42*
N_area_	1.72 (0.11)	1.34 (0.10)	6.53*	1.71 (0.11)	1.41 (0.10)	7.36**
N_mass_	34.2 (1.2)	33.0 (1.1)	0.20	43.8 (1.1)	26.8 (1.1)	113.49**
PNUE	120 (13)	124 (12)	0.00	107 (12)	131 (12)	1.68
LMA	54.9 (3.1)	44.3 (2.8)	4.83*	40.7 (3.0)	55.5 (2.9)	10.37**
Leaf thickness	0.019 (0.001)	0.016 (0.001)	18.25**	0.016 (0.001)	0.019 (0.001)	20.77**
Leaf density	2.86 (0.18)	2.70 (0.16)	0.13	2.49 (0.17)	2.98 (0.17)	2.15
**Biochemistry**						
P_sol_ (mass)	11.3 (1.0)	10.6 (1.0)	0.08	17.20 (0.99)	6.80 (0.97)	46.58**
P_sol_ (area)	0.55 (0.09)	0.41 (0.08)	1.35	0.67 (0.09)	0.36 (0.09)	17.25**
P_sol_ (% N)	34.8 (2.5)	39.0 (2.4)	1.44	45.3 (2.5)	31.3 (2.4)	16.69**
P_mem_ (mass)	11.63 (0.98)	9.05 (0.93)	7.34**	11.96 (0.95)	9.26 (0.93)	5.81*
P_mem_ (area)	0.64 (0.07)	0.36 (0.07)	7.78**	0.49 (0.07)	0.51 (0.07)	0.09
P_mem_ (% N)	43.4 (2.2)	36.6 (2.1)	5.10*	33.8 (2.1)	44.1 (2.1)	12.26**
P_cw_ (mass)	2.22 (0.19)	1.71 (0.18)	5.58*	2.11 (0.19)	1.87 (0.18)	1.16
P_cw_ (area)	0.12 (0.01)	0.07 (0.01)	7.76**	0.08 (0.01)	0.10 (0.01)	1.59
P_cw_ (% N)	8.88 (0.65)	7.73 (0.61)	1.15	6.01 (0.63)	9.84 (0.61)	19.05**
A/P_sol_	41.7 (7.1)	44.9 (6.6)	0.02	25.3 (6.9)	55.3 (6.6)	11.53**
P_sol_/P_mem_	1.00 (0.18)	1.33 (0.17)	3.24	1.71 (0.18)	0.81 (0.17)	15.90**
P_total_ (mass)	25.1 (1.5)	21.4 (1.4)	6.81*	31.3 (1.5)	17.9 (1.4)	45.68**
P_total_ (area)	1.31 (0.15)	0.85 (0.14)	14.43**	1.24 (0.15)	0.97 (0.14)	10.76**
AA (mass)	0.78 (0.09)	0.93 (0.08)	2.03	1.26 (0.08)	0.59 (0.08)	65.13**
AA (% N)	2.60 (0.30)	3.88 (0.28)	8.49**	3.43 (0.29)	3.12 (0.28)	7.08*
TNA (mass)	2.40 (0.16)	2.42 (0.15)	1.38	3.35 (0.16)	1.78 (0.15)	57.94**
TNA (% N)	8.49 (0.68)	9.17 (0.64)	0.49	9.44 (0.66)	8.42 (0.65)	1.51
NH_4_^+^ (mass)	0.11 (0.02)	0.21 (0.02)	3.64	0.05 (0.02)	0.23 (0.02)	71.50**
NH_4_^+^ (% N)	0.44 (0.20)	1.67 (0.19)	9.01**	0.19 (0.19)	1.63 (0.19)	126.53**
NO_3_^−^ (mass)	0.36 (0.11)	0.42 (0.11)	0.00	0.59 (0.11)	0.25 (0.11)	1.51
NO_3_^−^ (% N)	1.44 (0.45)	2.00 (0.44)	1.56	1.87 (0.45)	1.62 (0.43)	2.30
Chl (mass)	780 (76)	856 (62)	0.70	1043 (74)	712 (62)	11.58**
Chl (area)	40.9 (5.2)	35.9 (4.2)	0.66	41.1 (5.0)	36.6 (4.2)	1.02

Statistical effects of ‘origin’ (native/invasive) and ‘legume’ (legume/non-legume) are F-ratios with statistically significant values denoted by * *P*<0.05 and ** *P*<0.01. For both analyses, ‘species’ (df = 8) was nested within ‘origin’ (df = 1) or ‘legume’ (df = 1). Trait abbreviations: A_area_, photosynthetic rate ( µmol CO_2_ m^–2^ leaf s^–1^); A_mass_, photosynthetic rate (nmol g^−1^ s^−1^); V_max_, maximum rate of carboxylation ( µmol CO_2_ m^–2^ leaf s^−1^); J_max_, maximum electron transport rate ( µmol electrons m^−2^ s^−1^); φ_PSII_, effective quantum yield of PSII, (ΔF/F_m_’); N_area_, leaf N (g N m^−2^); N_mass_, leaf N (mg N g^−1^); PNUE, photosynthetic nitrogen use efficiency ( µmol CO_2_ mol^–1^ N s^–1^); LMA, leaf mass per area (g m^−2^); Leaf thickness (mm); Leaf density (g cm^−3^); P_sol_, soluble protein (mg N g^−1^ and g N m^−2^); P_mem_, membrane-bound protein (mg N g^−1^ and g N m^−2^); P_cw_, cell-wall protein (mg N g^−1^ and g N m^−2^); AA, amino acid (mg N g^−1^); TNA, total nucleic acid (mg N g^−1^); NH_4_^+^, ammonium (mg N g^−1^); NO_3_^−^, nitrate (mg N g^−1^); Chl, Chorophyll A + B (ug g^−1^ and mg m^−2^);% N, percent of total leaf N.

These results were not confounded with differences in LMA across native and invasive species (data not shown). At a given LMA, most traits showed differences between native and invasive species: for N_area_ and cell-wall protein concentration because the slopes of the trait against LMA differed between native and invasive species (*P*-values = 0.02–0.03) and for membrane-bound protein concentration and NH_4_^+^ content per area the slopes were statistically similar but the intercept was higher for native or invasive species (*P*-values = 0.01–0.03). For one trait, the percent of total leaf N allocated to amino acids, native and invasive showed the same relationship with LMA (slope *P* = 0.58; intercept *P* = 0.97), so the differences in that case were explained by LMA.

**Figure 2:**
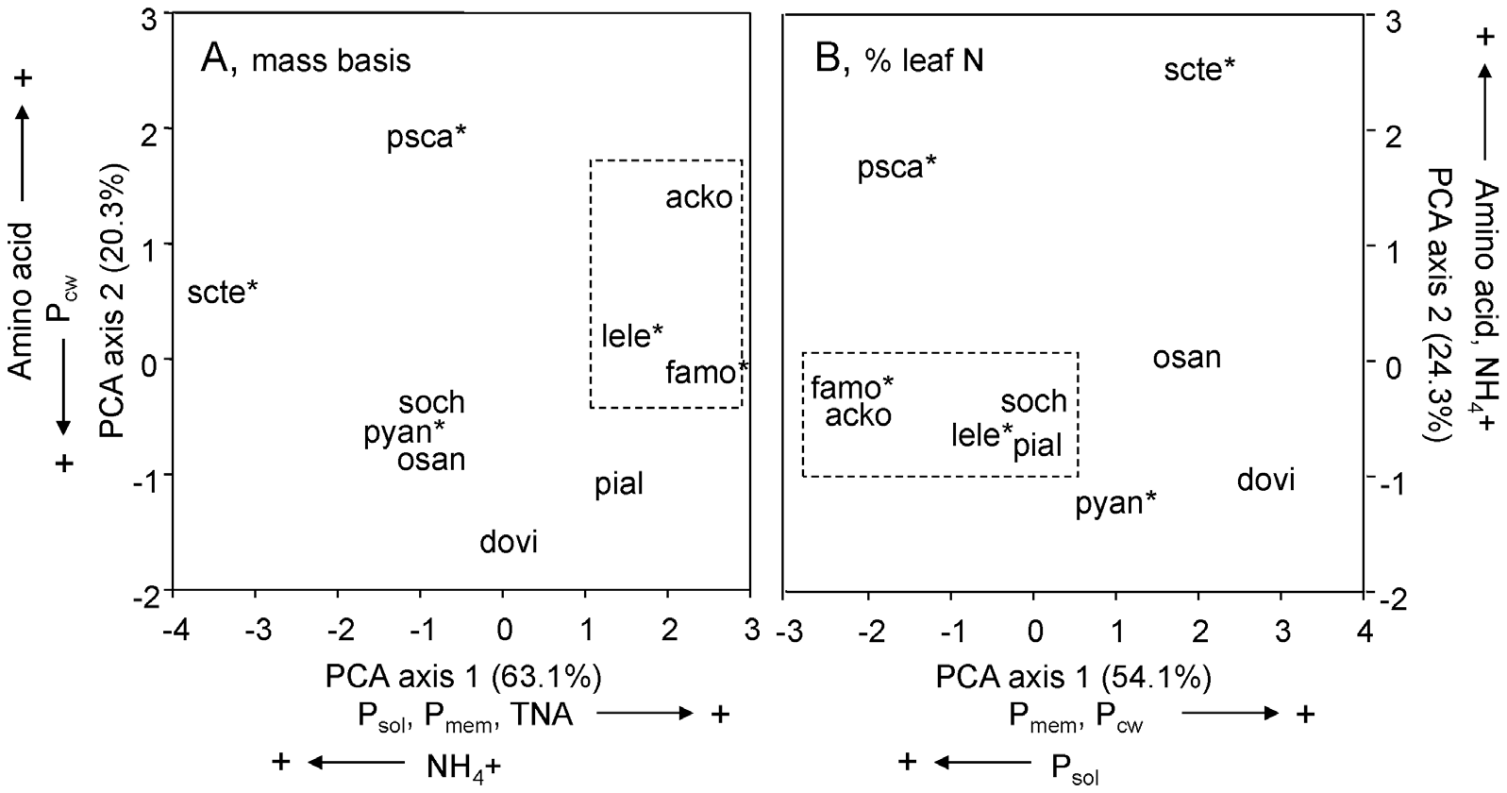
Results of a principal components analysis for six N fractions from five native and five invasive (*) species grown at 300 µmol photon m^−2^ s^−1^ on a mass basis (A) and as a percentage of total leaf N (B). Legumes tended to cluster together (dashed square) in both panels. Two invasive species differed from the other species: *Psidium* had lower membrane-bound and cell-wall protein and higher amino acid concentration whereas *Schinus* had high NH_4_^+^ concentration. Species abbreviations are provided in Figure 1.

A multivariate analysis showed that two invasive species were biochemically distinct from all other species (Figure 2). *Psidium* tended to have high concentrations of amino acids and NH_4_^+^, and low concentrations of membrane-bound and cell-wall proteins. *Schinus* differed from other species in its high NH_4_^+^ concentration and low nucleic acid concentration.

### Variation in nitrogen partitioning between legumes and non-legumes

Legumes generally had higher total protein and soluble protein concentrations and content per area (Table 1). Non-legumes allocated a larger fraction of leaf N to membrane-bound and cell-wall protein fractions than did legumes (Table 1). Legumes had substantially higher ratios of soluble to membrane-bound protein than non-legumes (1.71 vs. 0.81, Table 1). Total nucleic acids and amino acids were higher in legumes than non-legumes on both a mass and area basis (Table 1, Table S1). Leaf NO_3_^−^ concentration was significantly higher in two legumes, *Leucaena* and *Sophora*, than in all other species (Figure 1, Table S1).

The multivariate analysis showed that legumes clustered together with high soluble protein and nucleic acid concentration when expressed as a percentage of total leaf N (Figure 2). The exception was *Sophora*, one of the native legumes, which clustered with the majority of non-legumes when traits were expressed on a mass basis.

### Functional significance of N partitioning

Biochemical traits were more strongly inter-correlated on a mass basis than an area basis (Table 2). On a mass basis, soluble protein was strongly positively correlated with most biochemical fractions, with the exception of NH_4_^+^, with which it was negatively correlated. Few biochemical fractions correlated with physiological function or leaf morphological traits. Membrane-bound protein correlated positively with light use efficiency (φ_PSII_), although this did not translate into higher rates of photosynthesis (Table 3). Species with high soluble and membrane-bound protein concentration tended to have higher rates of photosynthesis (A_mass_), but this was not statistically significant. Leaf NH_4_^+^ was negatively correlated with all four measures of physiological function (A, V_max_, J_max_, φ_PSII_). Amino acid and chlorophyll concentrations (area basis) were negatively correlated with PNUE. Lastly, species with high LMA and density had higher area-based cell wall protein concentration, but lower mass-based soluble protein, nucleic acid and chlorophyll concentration (Table 3).

**Table 2:** Correlation coefficients (r) for relationships among leaf N fractions for five native and five invasive species grown at 300 µmol photons m^−2^ s^−1^ and high nutrient availability (n = 10 species).

	P_sol_	P_mem_	P_cw_	AA	TNA	NH_4_^+^	NO_3_^−^	Chl	N
P_sol_	–	0.78**	0.50	0.71*	0.88**	−0.45	−0.04	0.76*	0.75*
P_mem_	0.79**	–	0.84**	0.40	0.72*	−0.20	0.06	0.62+	0.68*
P_cw_	0.63*	0.85**	–	0.30	0.59+	0.06	−0.10	0.44	0.56+
AA	0.75*	0.40	0.30	–	0.70*	−0.09	0.19	0.68*	0.72*
TNA	0.93**	0.76*	0.72*	0.75*	–	−0.43	−0.07	0.66+	0.53
NH_4_^+^	−0.65*	−0.57+	−0.54	−0.38	−0.59+	–	0.11	0.13	0.01
NO_3_^−^	0.29	0.29	−0.05	0.30	0.25	−0.14	–	0.50	0.16
Chl	0.86**	0.64+	0.35	0.63+	0.78*	−0.25	0.70*	–	0.74*
N	0.93**	0.68*	0.41	0.74*	0.77*	−0.63+	0.40	0.86**	–

Correlations between area- and mass-based traits are on the upper half and lower half of the table, respectively. Correlations were performed on log-transformed data. Significant correlations at *P*<0.10 (+), *P*<0.05 (*) and *P*<0.01 (**) are shown. Trait abbreviations are given in Table 1.

**Table 3:** Correlation coefficients (r) for relationships among leaf biochemical, physiological and morphological traits for five native and five invasive species grown at 300 µmol photons m^−2^ s^−1^ and high nutrient availability (n = 10 species).

	A_area_	A_mass_	V_max_	J_max_	φ_PSII_	PNUE	LMA	Density	Thick
P_sol_ (mass)	0.18	0.53	0.33	0.06	0.49	−0.05	−0.59+	−0.54	−0.18
P_sol_ (area)	0.14	0.10	0.44	0.16	0.56+	−0.41	−0.02	0.05	0.05
P_sol_ (% N)	−0.07	0.48	0.04	−0.01	0.13	−0.05	−0.66*	−0.50	−0.43
P_mem_ (mass)	0.43	0.53	0.50	0.14	0.71*	0.15	−0.42	−0.49	0.10
P_mem_ (area)	0.33	−0.04	0.54	0.23	0.64*	−0.25	0.27	0.12	0.37
P_mem_ (% N)	0.29	−0.12	0.16	0.15	0.11	0.29	0.37	0.25	0.31
P_cw_ (mass)	0.39	0.34	0.44	0.04	0.67*	0.14	−0.24	−0.38	0.27
P_cw_ (area)	0.18	−0.67	0.36	0.11	0.43	−0.31	0.58+	0.35	0.55+
P_cw_ (% N)	−0.02	−0.50	−0.13	−0.04	−0.23	0.09	0.63*	0.50	0.36
AA (mass)	0.01	0.19	0.15	0.06	0.23	−0.33	−0.30	−0.28	−0.10
AA (area)	−0.09	−0.23	0.16	0.11	0.17	−0.64*	0.19	0.15	0.10
AA (% N)	−0.30	−0.24	−0.19	0.01	−0.30	−0.47	0.07	0.12	−0.12
TNA (mass)	0.10	0.47	0.21	−0.02	0.39	0.00	−0.59+	−0.68*	0.09
TNA (area)	0.03	0.03	0.29	0.07	0.40	−0.33	−0.05	−0.26	0.40
TNA (% N)	−0.29	0.08	−0.25	−0.20	−0.22	−0.01	−0.37	−0.57+	0.30
NH_4_^+^ (mass)	−0.57+	−0.58+	−0.81**	−0.67*	−0.71*	−0.24	0.31	0.23	0.21
NH_4_^+^ (area)	−0.53	−0.78**	−0.66*	−0.50	−0.64*	−0.43	0.63*	0.52	0.33
NH_4_^+^ (% N)	−0.52	−0.64*	0.72*	−0.48	−0.74*	−0.19	0.45	0.40	0.18
NO_3_^−^ (mass)	−0.06	0.28	0.18	0.14	−0.06	0.00	−0.43	−0.42	−0.11
NO_3_^−^ (area)	−0.18	−0.21	0.18	0.24	−0.22	−0.37	0.14	0.09	0.10
NO_3_^−^ (% N)	−0.28	−0.20	−0.19	0.01	−0.54	−0.05	0.07	0.13	−0.13
Chl (mass)	−0.28	0.31	−0.05	−0.27	0.01	−0.16	−0.62+	−0.69*	0.06
Chl (area)	−0.47	−0.49	0.00	−0.16	−0.08	−0.81**	0.30	0.10	0.45

Correlations were performed on log-transformed data. Significant correlations at *P*<0.10 (+), *P*<0.05 (*) and *P*<0.01 (**) are shown. Trait abbreviations are given in Table 1.

## Discussion

Intra-leaf N partitioning should reflect trade-offs on the leaf economics spectrum with faster growing species allocating more N to metabolic processes at the expense of structure. Thus, we hypothesized that invasive species, which are generally located on the “high-return” end of the leaf economics spectrum, would have lower LMA and higher A relative to native species, with greater allocation to leaf N pools associated with photosynthesis and growth. In support of this idea, across all species, LMA was negatively correlated with soluble protein, total nucleic acid and chlorophyll content and positively correlated with cell wall protein. Invasive species as a group did have lower LMA and cell wall protein with higher amino acid content, consistent with allocation to growth at the expense of structure. However, our hypothesis that invasive species would allocate more resources to carbon assimilation and growth at the expense of structure was only partially supported. Photosynthetic rates were either similar (mass-based) or higher (area-based) in native species relative to invasive species, soluble protein did not differ between groups, and native species had higher amounts of total N and membrane-bound protein.

Our finding that native and invasive species did not differ in the allocation of N to soluble protein contrasts with previously published results that invasive populations of *Ageratina adenophora* allocated more N to soluble protein at the expense of cell-wall protein compared with native populations [18]. Despite no difference in soluble protein between native and invasive species, native species had higher A_area_ and V_max_ compared with invasive species and this suggests that Rubisco content or activity may have been higher in native species. Our soluble protein fraction includes Rubisco and many other proteins, but Rubisco was not directly measured in this study.

The low irradiance used in our experiment (which reflects actual growing conditions of seedlings in Hawaiian forest understory) resulted in relatively even allocation of N to light harvesting (e.g., membrane-bound protein) and carbon assimilation (e.g. soluble protein) functions. Our data matched theoretical estimates modeled from gas exchange data, indicating that shade leaves should have a 1∶1 ratio of soluble and membrane-bound protein whereas sun leaves should have 2–3 times higher soluble than membrane-bound protein [12], [31]; in our light-limited growth environment, we found roughly a 1∶1 ratio of soluble to membrane-bound protein with no significant difference between native and invasive species. However, native species allocated more N to membrane-bound protein (43.4% of total N) than invasive species (36.6% of total N). Because the membrane-bound protein fraction includes pigment-protein complexes which promote effective light harvesting and electron transport, higher amounts of membrane-bound protein in native species may have contributed to the higher photosynthetic rates (A_area_, J_max_) under our light-limited growth conditions. While membrane-bound protein was higher in native species relative to invasive species, chlorophyll content was similar and this suggests that the membrane-bound protein fraction may represent protein that may or may not be involved in carbon assimilation.

Two invasive species (*Falcataria, Psidium*) and one native species (*Acacia*) displayed high soluble protein concentration and, consequently, low photosynthetic rates per unit soluble protein (A/P_sol_), which conflicts with predictions of optimal N partitioning models. These models, run across irradiance environments, suggest that shade leaves should allocate less N to soluble protein as photosynthesis becomes limited by light harvesting rather than carbon assimilation at low irradiance [54]. Although a high production of soluble protein seems like a wasteful use of N at low irradiance, it is possible that higher protein and amino acid concentration in *Acacia, Falcataria* and *Psidium* represented N storage [55], [56] and this could allow rapid growth in a heterogeneous light environment (e.g., tree gaps, forest edges), although the rate of amino acid production rather than pool size is more commonly related to relative growth rate. A recent study supports the idea that *Acacia* has high phenotypic plasticity in response to light availability [57] which suggests that stored N may be useful for rapid growth under changing light conditions.

Our hypotheses for N allocation outside of protein were partially supported. We predicted that invasive species would allocate more N to amino acids and nucleic acids which are required in greater amounts in rapidly growing tissues [21], [22]. While invasive species allocated a larger percentage of leaf N to amino acids compared with native species, total nucleic acid concentration did not vary between groups. Strong species-level variation was evident, and thus differences among native and invasive species for a given N fraction were often driven by a single species. Considering all the biochemical fractions, two invaders were distinct from all other species. *Psidium* tended to have high amino acid concentration, high NH_4_^+^ concentration, and low membrane-bound and cell-wall protein concentration while *Schinus* differed from other species primarily in its high NH_4_^+^ concentration and low nucleic acid concentration. These findings at the biochemical level are analogous to other studies that showed great trait variation within groups of native and/or invasive species: such a pattern has been reported for leaf nutrient concentrations, LMA, canopy height, seed mass, relative growth rate, and water use [2], [58]–[60]. Thus, the results of comparisons of native and invasive species will depend on the particular species considered. The overall average differences between natives and invasives are instructive, but species- level comparisons provide higher resolution that might indicate outcomes of these trait differences at the plant level. Thus, the pertinent question is: what are the functional consequences of this inter-specific variation in N partitioning?

The partitioning patterns for *Psidium* were consistent with general expectations concerning invasive species: more N was allocated to growth (amino acids) at the expense of structure (cell-wall protein). *Psidium* may succeed in dense understories by increasing amino acid production for transport out of the leaf to fuel the production of new structures. Increasing whole-plant leaf area will maximize light interception in Hawaii's light-limited mesic forests. Our results concur with those of Niinemets *et al.* [28] who found that an invasive species (*Rhododendron ponticum*) diluted leaf N at a high fertility, forested site, making more leaves rather than increasing leaf N concentration. The authors concluded that, at a plant level, greater light interception may be more advantageous than a higher Chl/N ratio in a forest understory.

Conversely, the patterns in *Schinus*, particularly its high levels of NH_4_^+^, suggested stress rather than optimal N partitioning. NH_4_^+^ accumulation in leaves is toxic and under normal conditions leaf NH_4_^+^ levels are relatively constant in plants. Differences in leaf NH_4_^+^ among species may indicate a decrease in amino acid production most likely resulting from stress associated with low irradiance. Low total protein concentration in *Schinus* drove the overall higher total protein concentration of natives relative to invasives. While *Schinus* seedlings often recruit within dense patches of established adult plants, our results suggest that this species spreads by a mechanism other than superior leaf biochemistry, possibly vegetative reproduction into high light environments (J Funk, personal observation).

We found that nitrogen-fixation strongly influenced intra-leaf N allocation. Legumes had higher total protein content, higher soluble protein content, and a higher ratio of soluble to membrane-bound protein relative to non-legumes. As noted above, this pattern of N allocation may be disadvantageous in a light-limited habitat where membrane-bound protein will increase light harvesting. However, we should note that A was not correlated with membrane-bound protein across species in this study. A study of nitrogen-fixing and non-fixing plant species in the United States found that nitrogen-fixing species tended to be less shade tolerant than non-fixers [61]; thus, high soluble protein in these species may be beneficial in open canopies or for rapid growth when gaps appear in the canopy. As discussed above, legumes may store N as soluble protein for use when light becomes available in the understory.

We acknowledge limitations in our study design. First, we were unable to find seeds for co-occurring, phylogenetically related pairs of native and invasive species or pairs of invasive and noninvasive exotic species. Because more closely related taxa share similar trait values, phylogenetic comparative designs minimize trait differences associated with comparing unrelated species and disparate life forms. Thus, future biochemical studies should include more taxonomically based species comparisons. A second limitation was that we did not directly measure relative growth rates of our species. While many studies infer inter-specific differences in hard-to-measure processes like growth from easy-to-measure traits like LMA based on established leaf economics spectrum relationships, e.g., [5], we assumed that our invasive species were located closer to the “high return” end of the leaf economics spectrum relative to native species based on differences in LMA. Consequently, we were unable to directly link species differences in amino acid and nucleic acid content to relative growth rates. Third, our study was conducted at high N availability and we may expect to see greater differences in N allocation, and stronger differences between native and invasive species in N partitioning and leaf-level traits, under low N conditions. Our experiment initially included a low N treatment but high mortality resulted in too few replicates for our final analyses.

Lastly, our estimates of protein fractions for the three species with leaf phenolic content greater than 12% (*Pipturus, Psidium, Schinus*) and low percent of N recovery for some species added error to the data and, consequently, our interpretation. In particular, we only recovered 38% of total leaf N from *Schinus* which, along with *Psidium*, was an outlier with respect to several N pools. *Schinus* is the only species we surveyed that contains leaf resin which may have interfered with extraction and quantification of soluble protein. Underestimates of soluble protein in *Schinus* and *Psidium* would result in lower amounts of NH_4_^+^ and amino acids than we report here, making the two species more similar in biochemistry to the other invasive species measured. Additionally, *Sophora* had lower soluble protein content relative to other legumes, and low N recovery (64%) for this species may reflect an underestimate of soluble protein.

Despite these limitations, our study is the first to examine intra-leaf N partitioning in a suite of native and invasive species. Our data suggest that invasive species employ a wide range of mechanisms in N allocation. For example, some invasive species (particularly *Falcataria* and *Leucaena*) may succeed as invaders in low-light environments by storing N as protein and amino acids that can be used when high-light conditions become available. Conversely, our results suggest that one invader (*Psidium*) may succeed by allocating N to growth at the expense of higher leaf-level carbon assimilation. More studies are needed to confirm these patterns across a larger number of native and invasive species and to evaluate the potential importance of biochemistry, in combination with other factors (e.g., clonality, enemy release, seed dispersal), in contributing to the success of invasive species.

## Supporting Information

Table S1 **Trait values for five native and five invasive species.**
(PDF)

Methods S1 **Correction of protein yields for interference by polyphenols.**
(PDF)

Results S1 **Nitrogen recovery in biochemical fractions.**
(PDF)

## References

[1] Millennium Ecosystem Assessment (2005) Ecosystems and Human Well-Being: Biodiversity Synthesis. Washington, DC:World Resources Institute.

[2] Leishman MR, Thomson VP, Cooke J (2010) Native and exotic invasive plants have fundamentally similar carbon capture strategies. Journal of Ecology 98: 28–42.

[3] Leishman MR, Haslehurst T, Ares A, Baruch Z (2007) Leaf trait relationships of native and invasive plants: community- and global-scale comparisions. New Phytologist 176: 635–643. 17822409 10.1111/j.1469-8137.2007.02189.x

[4] Penuelas J, Sardans J, Llusia J, Owen SM, Carnicer J, et al (2010) Faster return on 'leaf economics' and different biogeochemical niche in invasive compared with native plant species. Global Change Biology 16: 2171–2185.

[5] Reich PB, Wright IJ, Lusk CH (2007) Predicting leaf physiology from simple plant and climate attributes: A global glopnet analysis. Ecological Applications 17: 1982–1988. 17974336 10.1890/06-1803.1

[6] Wright IJ, Westoby M (2000) Cross-species relationships between seedling relative growth rate, nitrogen productivity and root vs leaf function in 28 australian woody species. Functional Ecology 14: 97–107.

[7] Poorter L, Wright SJ, Paz H, Ackerly DD, Condit R, et al (2008) Are functional traits good predictors of demographic rates? Evidence from five neotropical forests. Ecology 89: 1908–1920. 18705377 10.1890/07-0207.1

[8] Shipley B (2002) Trade-offs between net assimilation and specific leaf area in determining relative growth rate: Relationship with daily irradiance. Functional Ecology 16: 682–689.

[9] Grotkopp E, Erskine-Ogden J, Rejmanek M (2010) Assessing potential invasiveness of woody horticultural plant species using seedling growth rate traits. Journal of Applied Ecology 47: 1320–1328.

[10] Daehler CC (2003) Performance comparisons of co-occurring native and alien invasive plants: implications for conservation and restoration. Annual Review of Ecology, Evolution and Systematics 34: 183–211.

[11] Field CB, Mooney HA (1986) The photosynthesis-nitrogen relationship in wild plants. In: Givnish TJ, editor. On the Economy of Plant Form and Function. Cambridge, England:Cambridge University Press. pp. 25–55.

[12] Evans JR (1989) Photosynthesis and nitrogen relationships in leaves of C3 plants. Oecologia 78: 9–19. 28311896 10.1007/BF00377192

[13] Warren CR, Adams MA (2001) Distribution of N, Rubisco and photosynthesis in *Pinus pinaster* and acclimation to light. Plant, Cell and Environment 24: 597–609.

[14] Harrison MT, Edwards EJ, Farquhar GD, Nicotra AB, Evans JR (2009) Nitrogen in cell walls of sclerophyllous leaves accounts for little of the variation in photosynthetic nitrogen-use efficiency. Plant, Cell and Environment 32: 259–270. 10.1111/j.1365-3040.2008.01918.x 19054350

[15] Onoda Y, Hikosaka K, Hirose T (2004) Allocation of nitrogen to cell walls decreases photosynthetic nitrogen-use efficiency. Functional Ecology 18: 419–425.

[16] Takashima T, Hikosaka K, Hirose T (2004) Photosynthesis or persistence: nitrogen allocation in leaves of evergreen and deciduous *Quercus* species. Plant, Cell and Environment 27: 1047–1054.

[17] Reiter WD (1998) The molecular analysis of cell wall components. Trends in Plant Science 3: 27–32.

[18] Feng Y, Lei Y-B, Wang Y-P, Callaway RM, Valiente-Banuet A, et al (2009) Evolutionary tradeoffs for nitrogen allocation to photosynthesis versus cell walls in an invasive plant. Proceedings of the National Academy of Sciences 106: 1853–1856. 10.1073/pnas.0808434106 PMC2644127 19171910

[19] Hikosaka K, Shigeno A (2009) The role of Rubisco and cell walls in the interspecific variation in photosynthetic capacity. Oecologia 160: 443–451. 19288136 10.1007/s00442-009-1315-z

[20] Chapin FS III, Kedrowski RA (1983) Seasonal changes in nitrogen and phosphorus fractions and autumn retranslocation in evergreen and deciduous taiga trees. Ecology 64: 376–391.

[21] Chapin FS III, Shaver GR, Kedrowski RA (1986) Environmental controls over carbon, nitrogen and phosphorus fractions in *Eriophorum vaginatum* in Alaskan tussock tundra. Journal of Ecology 74: 167–195.

[22] Lambers H, Poorter H (1992) Inherent variation in growth rate between higher plants: a search for physiological causes and ecological consequences. Advances in Ecological Research 23: 187–261.

[23] Brix H (1981) Effects of fertilizer source and application rates on foliar nitrogen concentration, photosynthesis, and growth of Douglas-fir. Canadian Journal of Forest Research 11: 775–780.

[24] Walters MB, Reich PB (1989) Response of *Ulmus americana* seedlings to varying nitrogen and water status. 1. Photosynthesis and growth. Tree Physiology 5: 159–172. 14972984 10.1093/treephys/5.2.159

[25] Wendler R, Millard P (1996) Impacts of water and nitrogen supplies on the physiology, leaf demography and nitrogen dynamics of *Betula pendula* . Tree Physiology 16: 153–159. 14871759 10.1093/treephys/16.1-2.153

[26] Coleman MD, Dickson RE, Isebrands JG (1998) Growth and physiology of aspen supplied with different fertilizer addition rates. Physiologia Plantarum 103: 513–526.

[27] Warren CR, Chen ZL, Adams MA (2000) Effect of N source on concentration of Rubisco in *Eucalyptus diversicolor*, as measured by capillary electrophoresis. Physiologia Plantarum 110: 52–58.

[28] Niinemets U, Valladares F, Ceulemans R (2003) Leaf-level phenotypic variability and plasticity of invasive *Rhododendron ponticum* and non-invasive *Ilex aquifolium* co-occurring at two contrasting European sites. Plant, Cell and Environment 26: 941–956. 10.1046/j.1365-3040.2003.01027.x 12803621

[29] Funk JL, Jones CG, Lerdau MT (2007) Leaf- and shoot-level plasticity in response to varying nutrient and water availabilities. Tree Physiology 27: 1731–1739. 17938104 10.1093/treephys/27.12.1731

[30] Vitousek PM, Walker LR, Whiteaker LD, Matson PA (1993) Nutrient limitations to plant growth during primary succession in Hawaii Volcanoes National Park. Biogeochemistry 23: 197–215.

[31] Terashima I, Evans JR (1988) Effects of light and nitrogen nutrition on the organization of the photosynthetic apparatus in spinach. Plant and Cell Physiology 29: 143–155. 10.1111/j.1365-3040.2005.01453.x 17080618

[32] Wagner WL, Herbst DR, Sohmer SH (1999) Manual of the Flowering Plants of Hawaii. Honolulu:University of Hawaii Press.

[33] Farquhar GD, von Caemmerer S, Berry JA (1980) A biochemical model of photosynthetic CO_2_ assimilation in leaves of C3 species. Planta 149: 78–90. 24306196 10.1007/BF00386231

[34] Wullschleger SD (1993) Biochemical limitations to carbon assimilation in C3 plants - a retrospective analysis of the A/Ci curves from 109 species. Journal of Experimental Botany 44: 907–920.

[35] Long SP, Bernacchi CJ (2003) Gas exchange measurements, what can they tell us about the underlying limitations to photosynthesis? Procedures and sources of error. Journal of Experimental Botany 54: 2393–2401. 14512377 10.1093/jxb/erg262

[36] Lichtenthaler HK, Buschmann C (2001) Chlorophylls and carotenoids: measurement and characterization by UV-VIS spectroscopy. Current Protocols in Food Analytical Chemistry. New York: John Wiley and Sons.pp. F4.3.1–F4.3.8.

[37] Isaacson T, Damasceno CMB, Saravanan RS, He Y, Catala C, et al (2006) Sample extraction techniques for enhanced proteomic analysis of plant tissues. Nature Protocols 1: 769–774. 17406306 10.1038/nprot.2006.102

[38] Loomis WD, Battaile J (1966) Plant phenolic compounds and the isolation of plant enzymes. Phytochemistry 5: 423–428.

[39] Warren CR (2000) Is photosynthesis related to concentrations of nitrogen and Rubsico in leaves of Australian native plants? Australian Journal of Plant Physiology 27: 407–416.

[40] Mao QZ, Watanabe M, Imori M, Kim YS, Kita K, et al (2012) Photosynthesis and nitrogen allocation in needles in the sun and shade crowns of hybrid larch saplings: Effect of nitrogen application. Photosynthetica 50: 422–428.

[41] Frak E, Le Roux X, Millard P, Dreyer E, Jaouen G, et al (2001) Changes in total leaf nitrogen and partitioning of leaf nitrogen drive photosynthetic acclimation to light in fully developed walnut leaves. Plant, Cell and Environment 24: 1279–1288.

[42] Warren CR, Adams MA (2004) Evergreen trees do not maximize instantaneous photosynthesis. Trends in Plant Science 9: 270–274. 15165557 10.1016/j.tplants.2004.04.004

[43] Evans JR (1996) Developmental constraints on photosynthesis: effects of light and nutrition. In: Baker NR, editor. Photosynthesis and the Environment. The Netherlands: Kluwer Academic Publishers. pp. 281–304.

[44] Evans JR, Poorter H (2001) Photosynthetic acclimation of plants to growth irradiance: the relative importance of specific leaf area and nitrogen partitioning in maximizing carbon gain. Plant, Cell and Environment 24: 755–767.

[45] Pons TL, Westbeek MHM (2004) Analysis of differences in photosynthetic nitrogen-use efficiency between four contrasting species. Physiologia Plantarum 122: 68–78.

[46] McGrath R (1972) Protein measurement by ninhydrin determination of amino acids released by alkaline hydrolysis. Analytical Biochemistry 49: 95–102. 4673189 10.1016/0003-2697(72)90245-x

[47] Sun S-W, Lin Y-C, Weng Y-M, Chen M-J (2006) Efficiency improvements on ninhydrin method for amino acid quantification. Journal of food composition and analysis 19: 112–117.

[48] Bronk DA, Lomas MW, Gilbert PM, Schukert KJ, Sanderson MP (2000) Total dissolved nitrogen analysis: comparisons between the persulfate, UV and high temperature oxidation methods. Marine Chemistry 69: 163–178.

[49] Purcell LC, King CA (1996) Total nitrogen determination in plant material by persulfate digestion. Agromony Journal 88: 111–113.

[50] Noctor G, Bergot GL, Mauve C, Thominet D, Lelarge-Trouverie C, et al (2007) A comparative study of amino acid measurement in leaf extracts by gas chromatography-time of flight-mass spectrometry and high performance liquid chromatography with fluorescence detection. Metabolomics 3: 161–174.

[51] Husted S, Hebbern CA, Mattsson M, Schjoerring JK (2000) A critical experimental evaluation of methods for determination of NH_4_ ^+^ in plant tissue, xylem sap and apoplastic fluid. Physiologia Plantarum 109: 167–179.

[52] Leleu O, Vuylsteker C (2004) Unusual regulatory nitrate reductase activity in cotyledons of *Brassica napus* seedlings: enhancement of nitrate reductase activity by ammonium supply. Journal of Experimental Botany 55: 815–823. 14990621 10.1093/jxb/erh088

[53] Warton DI, Wright IJ, Falster DS, Westoby M (2006) Bivariate line-fitting methods for allometry. Biological Reviews 81: 259–291. 16573844 10.1017/S1464793106007007

[54] Hikosaka K, Terashima I (1995) A model of the acclimation of photosynthesis in the leaves of C3 plants to sun and shade with respect to nitrogen use. Plant, Cell and Environment 18: 605–618.

[55] Nasholm T, Edfast A-B, Ericsson A, Norden L-G (1994) Accumulation of amino acids in some boreal forest plants in response to increased nitrogen availability. New Phytologist 126: 137–143.

[56] Warren CR, Adams MA (2000) Capillary electrophoresis for the determination of major amino acids and sugars in foliage: application to the nitrogen nutrition of sclerophyllous species. Journal of Experimental Botany 51: 1147–1157. 10948242 10.1093/jexbot/51.347.1147

[57] Funk JL (2008) Differences in plasticity between invasive and native plants from a low resource environment. Journal of Ecology 96: 1162–1174.

[58] Cavaleri MA, Sack L (2010) Comparative water use of native and invasive plants at multiple scales: a global meta-analysis. Ecology 91: 2705–2715. 20957964 10.1890/09-0582.1

[59] Ordonez A, Wright IJ, Olff H (2010) Functional differences between native and alien species: a global-scale comparison. Functional Ecology 24: 1353–1361.

[60] van Kleunen M, Weber E, Fischer M (2010) A meta-analysis of trait differences between invasive and non-invasive plant species. Ecology Letters 13: 235–245. 20002494 10.1111/j.1461-0248.2009.01418.x

[61] Menge DNL, DeNoyer JL, Lichstein JW (2010) Phylogenetic constraints do not explain the rarity of nitrogen-fixing trees in late-successional temperate forests. PLOS One 5: e12056 doi:12010.11371/journal.pone.0012056. 20700466 10.1371/journal.pone.0012056 PMC2917374

