# Supplementary material for: Differential Allocation to Photosynthetic and Non-Photosynthetic Nitrogen Fractions among Native and Invasive Species
Source: PLoS One. 2013 May 20;8(5):e64502. doi: 10.1371/journal.pone.0064502 (PMC3659119; doi:10.1371/journal.pone.0064502)
Supplement: Table S1 — Trait values for five native and five invasive species. (PDF) [file pone.0064502.s001.pdf]

**Table S1** – Trait values for five native and five invasive species grown at 300 μmol photon $m^{-2}$ $s^{-1}$ and high nutrient availability. Values are means with SE in parentheses. Trait values not connected by the same letter (A-F) are statistically different at *P* < 0.05. ANOVA results are presented in Table 2 in the manuscript. Species and trait abbreviations are provided below. Sample sizes for each species (n) are provided next to species names. (NA) denotes no data collected. [L] denotes legume.

| | **Native species** | | | | | **Invasive species** | | | | |
|---|---|---|---|---|---|---|---|---|---|---|
| | acko[L] (n=6) | dovi (n=6) | osan (n=6) | pial (n=3) | soch[L] (n=6) | famo[L] (n=6) | lele[L] (n=6) | psca (n=6) | pyan (n=6) | scte (n=3) |
| $A_{area}$ | 14.3 (1.4) AB | 10.9 (0.8) ABC | 17.5 (1.6) A | 10.5 (1.6) ABC | 9.4 (1.5) BC | 10.1 (1.1) BC | 13.6 (1.7) AB | 7.0 (0.7) C | 13.4 (1.3) AB | 8.3 (0.9) BC |
| $A_{mass}$ | 388 (59) AB | 132 (16) D | 269 (34) BCD | 384 (17) ABC | 214 (73) CD | 272 (47) BCD | 464 (56) A | 134 (16) D | 465 (116) A | 139 (26) CD |
| $V_{max}$ | 113 (10) A | 98.6 (10.0) AB | 120 (7) A | 66.3 (0.1) ABC | 106 (17) AB | 92.1 (12.1) AB | 116 (13) A | 48.6 (4.0) C | 77.8 (4.2) ABC | 58.8 (7.3) BC |
| $J_{max}$ | 187 (20) AB | 131 (9) BC | 229 (23) A | 108 (3) BC | 209 (29) AB | 134 (16) BC | 155 (22) ABC | 104 (5) C | 148 (11) ABC | 114 (13) BC |
| ΦPSII | 0.27 (0.03) A | 0.25 (0.02) AB | 0.26 (0.02) A | 0.19 (0.02) AB | 0.17 (0.03) AB | 0.25 (0.02) AB | 0.27 (0.03) A | 0.15 (0.02) B | 0.23 (0.02) AB | 0.15 (0.02) AB |
| $c_i$ | 200 (11) A | 192 (8) A | 223 (15) A | 212 (26) A | 188 (17) A | 210 (13) A | 195 (23) A | 215 (15) A | 255 (14) A | 232 (21) A |
| $N_{area}$ | 2.36 (0.45) A | 2.32 (0.37) A | 1.48 (0.09) AB | 0.86 (0.07) B | 1.56 (0.11) AB | 1.53 (0.19) AB | 1.38 (0.04) AB | 1.60 (0.09) AB | 1.03 (0.13) B | 1.17 (0.10) B |
| $N_{mass}$ | 58.5 (4.0) A | 26.3 (1.4) DEF | 22.6 (2.0) EF | 31.7 (0.7) BCDEF | 31.9 (2.9) CD | 37.7 (2.1) BC | 47.1 (2.4) AB | 29.9 (1.0) CDE | 30.5 (0.8) CDE | 19.6 (3.3) F |
| PNUE | 99.6 (19.4) ABCD | 72.3 (10.1) CD | 168 (19) AB | 170 (12) ABCD | 87.9 (20.6) BCD | 103 (20) ABCD | 139 (18) ABC | 62.1 (6.3) D | 215 (54) A | 99.0 (3.9) ABCD |
| LMA | 40.7 (5.8) BCDE | 87.4 (11.0) A | 66.6 (3.2) AB | 27.3 (2.9) CDE | 52.3 (7.3) BCDE | 40.5 (4.3) BCDE | 29.4 (1.0) E | 53.6 (2.7) ABCD | 33.8 (4.2) DE | 64.0 (12.4) ABC |
| $P_{sol}$ | 25.3 (2.8) A | 7.0 (2.8) CDE | 4.2 (1.2) DE | 12.0 (0.1) ABCD | 7.9 (1.9) CDE | 20.0 (2.3) AB | 15.6 (2.4) ABC | 8.6 (1.0) BCD | 6.7 (0.7) CDE | 2.3 (1.2) E |
| $P_{mem}$ | 13.4 (1.8) A | 14.6 (2.2) A | 7.8 (0.9) AB | 14.3 (2.4) A | 8.0 (1.0) AB | 11.8 (3.6) A | 14.6 (2.1) A | 7.0 (0.6) AB | 9.1 (1.7) AB | 2.8 (0.8) B |
| $P_{cw}$ | 2.11 (0.36) AB | 2.66 (0.46) A | 2.03 (0.25) AB | 2.92 (0.76) A | 1.38 (0.22) BC | 2.86 (0.69) A | 2.06 (0.22) AB | 1.32 (0.13) BC | 1.36 (0.26) BC | 0.94 (0.23) C |
| $A/P_{sol}$ | 19.0 (5.5) BCD | 30.5 (6.9) ABCD | 94.1 (23.5) A | 32.0 (1.7) ABCD | 32.6 (9.0) ABCD | 15.1 (3.3) D | 34.4 (7.5) ABCD | 16.5 (2.4) BD | 75.3 (23.6) AC | 83.4 (23.5) ABC |

| | | | | | | | | | | |
|---|---|---|---|---|---|---|---|---|---|---|
| $P_{sol}/P_{mem}$ | 2.16 (0.45) AB | 0.50 (0.18) C | 0.56 (0.16) C | 0.86 (0.15) BC | 0.94 (0.15) C | 2.48 (0.75) A | 1.24 (0.30) BC | 1.28 (0.19) BC | 0.89 (0.22) C | 0.75 (0.19) C |
| TNA | 3.71 (0.34) AB | 1.35 (0.20) DE | 1.33 (0.17) DE | 3.91 (0.80) AB | 1.68 (0.28) CDE | 5.14 (0.45) A | 2.88 (0.54) BC | 23.9 (0.14) BCD | 1.20 (0.25) EF | 0.50 (0.17) F |
| AA | 2.07 (0.36) A | 0.33 (0.08) CD | 0.52 (0.11) C | 0.51 (0.26) BCD | 0.47 (0.07) CD | 1.07 (0.14) AB | 1.42 (0.16) A | 1.72 (0.19) A | 0.22 (0.02) D | 0.24 (0.04) CD |
| $NH_4^+$ | 0.05 (0.01) DE | 0.18 (0.02) ABC | 0.07 (0.02) CDE | 0.17 (0.01) ABCD | 0.06 (0.01) DE | 0.03 (0.01) E | 0.07 (0.02) DE | 0.38 (0.05) A | 0.13 (0.01) BCD | 0.44 (0.19) AB |
| $NO_3^-$ | 0.23 (0.03) BC | 0.20 (0.03) BC | 0.18 (0.03) BC | 0.38 (0.04) ABC | 0.80 (0.58) ABC | 0.15 (0.06) C | 1.17 (0.21) A | 0.37 (0.06) AB | 0.23 (0.03) BC | 0.16 (0.06) BC |
| Chl | NA | 9.4 (0.7) ABC | 8.3 (0.2) C | 15.1 (2.0) AB | 8.2 (0.8) AB | 10.7 (0.7) AB | 10.2 (0.6) A | 8.8 (0.5) A | 9.4 (0.4) ABC | 8.3 (0.8) BC |

Trait abbreviations: $A_{area}$, photosynthetic rate (μmol $CO_2$ $m^{-2}$ leaf $s^{-1}$); $A_{mass}$, photosynthetic rate (nmol $g^{-1}$ $s^{-1}$); $V_{max}$, maximum rate of carboxylation (μmol $CO_2$ $m^{-2}$ leaf $s^{-1}$); $J_{max}$, maximum electron transport rate (μmol electrons $m^{-2}$ $s^{-1}$); $\Phi_{PSII}$, effective quantum yield of PSII, ($\Delta F/F_m'$); $c_i$, internal $CO_2$ concentration (ppm); $N_{area}$, leaf N (g N $m^{-2}$); $N_{mass}$, leaf N (mg N $g^{-1}$); PNUE, photosynthetic nitrogen use efficiency (μmol $CO_2$ $mol^{-1}$ N $s^{-1}$); LMA, leaf mass per area (g $m^{-2}$); $P_{sol}$, soluble protein (mg N $g^{-1}$); $P_{mem}$, membrane-bound protein (mg N $g^{-1}$); $P_{cw}$, cell wall protein (mg N $g^{-1}$); AA, amino acid (mg N $g^{-1}$); TNA, total nucleic acid (mg N $g^{-1}$); $NH_4^+$, ammonium (mg N $g^{-1}$); $NO_3^-$, nitrate (mg N $g^{-1}$); Chl, Chorophyll A +B (ug $g^{-1}$).

Species abbreviations are: *Acacia koa* (acko), *Dodonaea viscosa* (dovi), *Falcataria moluccana* (famo), *Leucaena leucocephala* (lele), *Osteomeles anthyllidifolia* (osan), *Pipturus albidus* (pial), *Psidium cattleianum* (psca), *Pyracantha angustifolia* (pyan), *Schinus terebinthifolius* (scte), and *Sophora chrysophylla* (soch).
