# Supplementary material for: Differential Allocation to Photosynthetic and Non-Photosynthetic Nitrogen Fractions among Native and Invasive Species
Source: PLoS One. 2013 May 20;8(5):e64502. doi: 10.1371/journal.pone.0064502 (PMC3659119; doi:10.1371/journal.pone.0064502)
Supplement: Methods S1 — Correction of protein yields for interference by polyphenols. (PDF) [file pone.0064502.s002.pdf]

Methods S1 – Correction of protein yields for interference by polyphenols

Inter-specific comparisons of protein yields are rare partially because of the difficulty in extracting proteins uniformly across species. Species have different amounts of phenolic compounds in their leaves and these compounds interfere with protein extraction by binding proteins. Many protein extraction protocols use polyvinylpyrrolidone (PVP) or polyvinylpolypyrrolidone (PVPP, or cross-linked insoluble PVP) to absorb polyphenols (Loomis & Battaile 1996, Warren 2000, Isaacson *et al.* 2006). However, by experimentally adding tannic acid (a particularly complex phenolic compound) to fresh leaves during extraction, we found that even large amounts of PVPP (4%) did not protect proteins from absorption by polyphenols. Powdered tannic acid was added as a percentage of the leaf fresh weight to fresh *Ficus benjamina*, which we found to have < 0.1% total phenolics. Protein content was determined following methods outlined in the manuscript.

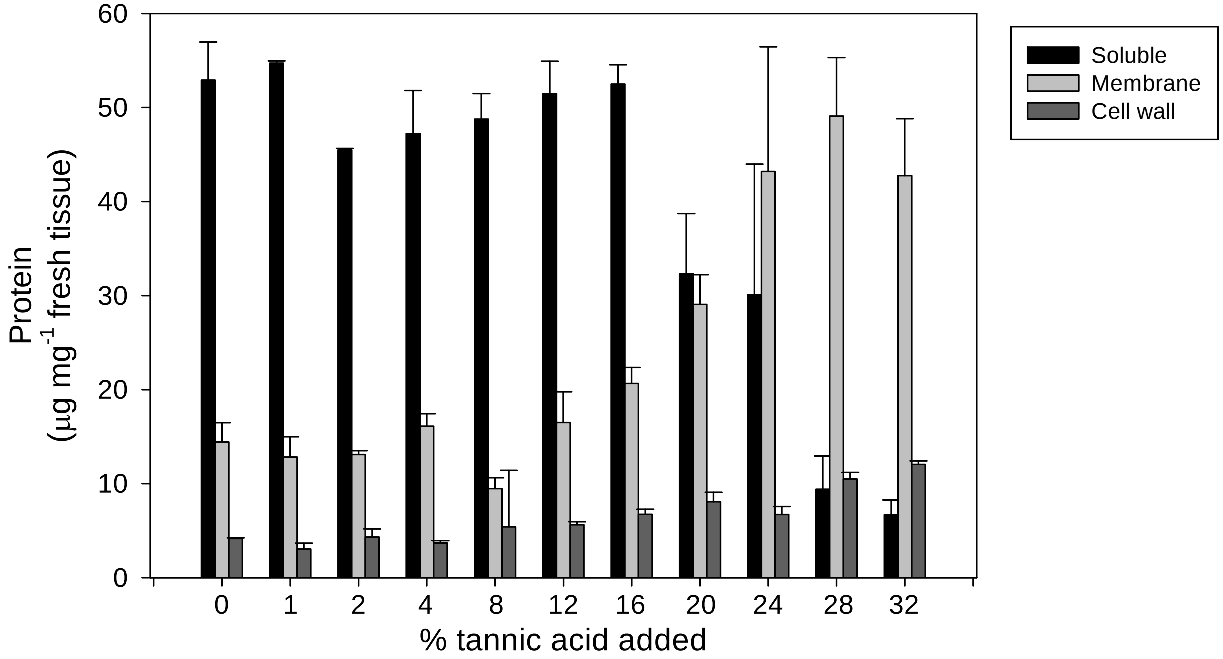

Based on the data we collected, we corrected protein data for species with total phenolic content > 12% dry leaf mass (*Pipturus, Psidium, Schinus*) for interference of polyphenols with protein determination. Equations were generated from the linear portion of curves below (> 12% phenolic content).

$WS_c = \frac{WS_i}{(TPC * -254 + 76.1)/49.8}$, where WS_c is the corrected water soluble protein content, WS_i is the initial protein content, and TPC is total phenolic content.

$MB_c = \frac{MB_i}{(TPC * 164.8 + 3.9)/13.2}$, where MB_c is the corrected membrane-bound protein content, MB_i is the initial membrane-bound protein content, and TPC is total phenolic content.

$CW_c = \frac{CW_i}{(TPC * 30 + 2.9)/4}$, where where CW_c is the corrected cell wall protein content, CW_i is the initial cell wall protein content, and TPC is total phenolic content.

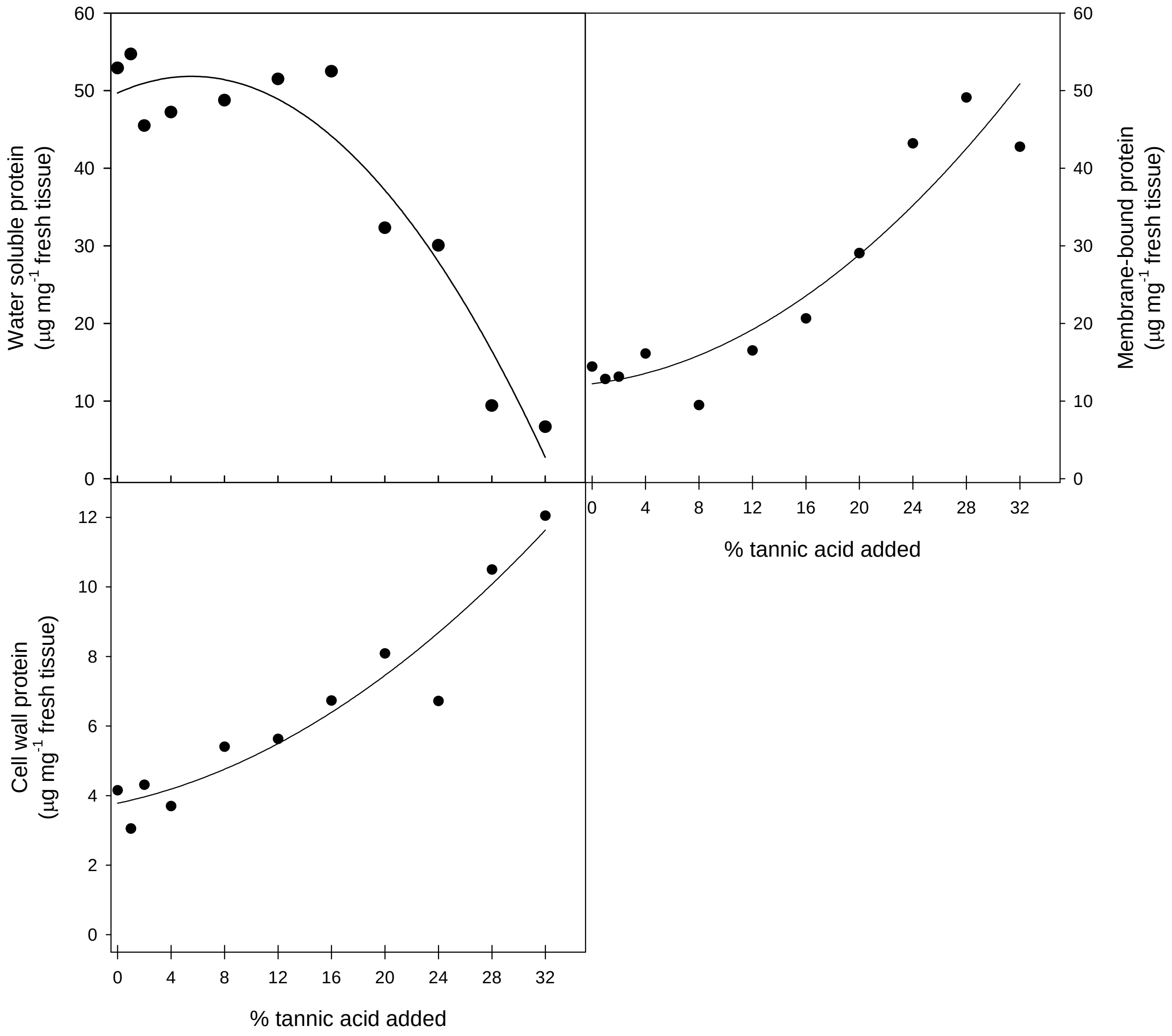

To determine total phenolic content, leaves were air-dried, ground, and analyzed using a Folin-Ciocalteu assay (AOAC 1950). Total phenolic data for all species are presented in the Table below, where * indicates invasive species.

**Species**	**Total phenolic s (% fresh weight)**
Acacia koa	1.55
Dodonaea viscosa	4.65
*Falcataria moluccana**	0.93
*Leucaena leucocephala**	5.10
Osteomeles anthyllidifolia	3.82

Pipturus albidus	0.00
*Psidium cattleianum**	22.25
*Pyracantha angustifolia**	12.40
*Schinus terebinthifolius**	22.56
Sophora chrysophylla	4.75

References

AOAC (1950). Official methods of analysis of the Association of Official Agricultural Chemists, 7th edition. Association of Official Agricultural Chemists, Washington, DC.

Isaacson T., Damasceno C.M.B., Saravanan R.S., He Y., Catala C., Saladie M. & Rose J.K.C. (2006). Sample extraction techniques for enhanced proteomic analysis of plant tissues. *Nature Protocols*, 1, 769-774.

Loomis W.D. & Battaile J. (1966). Plant phenolic compounds and the isolation of plant enzymes. *Phytochemistry*, 5, 423-428.

Warren C.R. (2000). Is photosynthesis related to concentrations of nitrogen and Rubsico in leaves of Australian native plants? *Australian Journal of Plant Physiology*, 27, 407–416.
