# Supplementary material for: Differential Allocation to Photosynthetic and Non-Photosynthetic Nitrogen Fractions among Native and Invasive Species
Source: PLoS One. 2013 May 20;8(5):e64502. doi: 10.1371/journal.pone.0064502 (PMC3659119; doi:10.1371/journal.pone.0064502)
Supplement: Results S1 — Nitrogen recovery in biochemical fractions. (PDF) [file pone.0064502.s003.pdf]

Results S1 – Nitrogen recovery in biochemical fractions

Across species, we recovered most of total leaf N in our seven fractions (soluble protein, membrane-bound protein, cell-wall protein, total nucleic acid, amino acid, NH_4^+, and NO_3^-). When we compared total leaf N concentration measured with an elemental analyzer (EA) with the sum of seven N fractions (fractions), the relationship was nearly 1:1 (see Figure below). Recovery varied across species (see Table below) with three species approaching 100% recovery and three species less than 70% recovery. Recovery did not vary in any systematic way. Recovery was not significantly different between invasive (72.3%) and native (84.7%) species groups ($t = 2.36$, $P = 0.42$) or between legume (83.3) and non-legume (75.3) species ($t = 2.31$, $P = 0.59$). Low N fraction recovery could be due to the presence of nitrogenous compounds not measured (e.g., alkaloids). For consistency across the results, we present the percentage of leaf N allocated to various N fractions using the value obtained by summing our seven fractions rather than that obtained by elemental analysis. Throughout, we use the term 'concentration' to refer to a parameter expressed per unit dry mass and 'content per area' for the area basis.

Species	% N (EA)	% N (fractions)	% Recovery
Acacia koa	5.85	4.69	80.2
Dodonaea viscosa	2.63	2.63	100.1
*Falcataria moluccana**	3.77	4.10	108.9
*Leucaena leucocephala**	4.71	3.78	80.2
Osteomeles anthyllidifolia	2.26	1.61	71.4
Pipturus albidus	3.17	3.42	107.9
*Psidium cattleianum**	2.99	2.17	72.7
*Pyracantha angustifolia**	3.05	1.89	61.9
*Schinus terebinthifolius**	1.96	0.74	37.9
Sophora chrysophylla	3.19	2.04	63.7

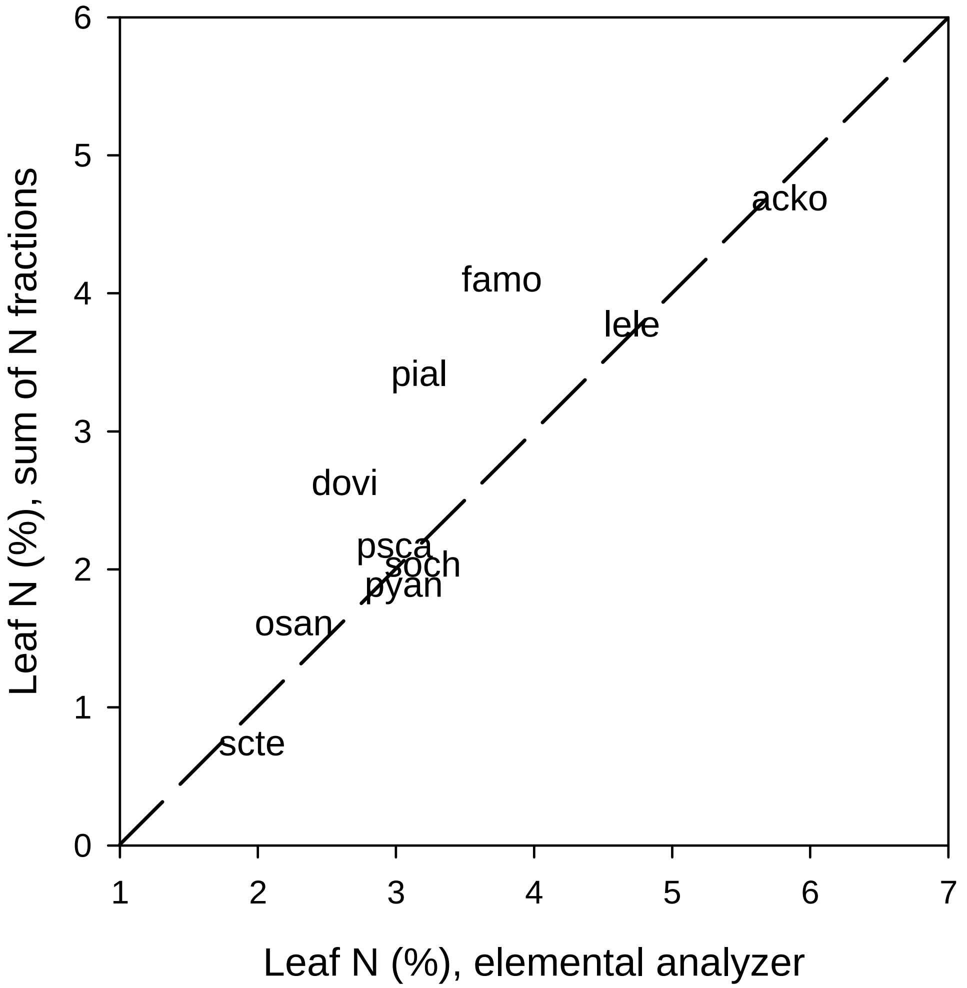
6
5
4
3
2
1
0
1
2
3
4
5
6
7
Leaf N (%), sum of N fractions
Leaf N (%), elemental analyzer
acko
famo
lele
pial
dovi
psca
soch
pyan
osan
scte
